# Prognostic significance of baseline skeletal muscle index and its dynamics in patients with metastatic breast cancer undergoing eribulin treatment

**DOI:** 10.1007/s10549-025-07827-y

**Published:** 2025-10-15

**Authors:** Masatsugu Amitani, Takaaki Oba, Ayaka Kitazawa, Ryoko Iji, Nami Kiyosawa, Shota Katsuyama, Hiroki Morikawa, Tatsunori Chino, Tadafumi Shimizu, Mayu Ono, Toshiharu Kanai, Ken-ichi Ito

**Affiliations:** Division of Breast and Endocrine Surgery, Department of Surgery, Shinshu University School of Medicine, 3-1-1 Asahi, Matsumoto, Nagano, Japan

**Keywords:** Skeletal muscle index, Metastatic breast cancer, Eribulin, Chemotherapy, Change in skeletal muscle index, Prognostic nutritional index

## Abstract

**Purpose:**

In breast cancer, a low skeletal muscle index (SMI) and prognostic nutritional index (PNI) negatively affect patient outcomes. However, the prognostic implications of changes in these values in patients with metastatic breast cancer (MBC) remain unclear. We evaluated the association between baseline levels and changes in SMI and PNI during eribulin treatment and patient outcomes.

**Methods:**

We retrospectively analyzed 67 patients with MBC treated with eribulin. SMI and PNI were assessed at baseline (pre-SMI, and pre-PNI) and at disease progression; changes from baseline were calculated. Patient outcomes were compared according to baseline status and direction of change.

**Results:**

SMI and PNI were not significantly correlated (*p* = 0.26, R = 0.02). High pre-SMI and high pre-PNI were associated with significantly improved overall survival (OS) (SMI; hazard ratio [HR] = 0.54, *p* = 0.04, PNI; HR = 0.33, *p* < 0.001). Patients with SMI gain during eribulin had longer OS than those with stable SMI or loss (HR = 0.48,* p* = 0.04), whereas PNI increase was not significantly associated with OS (HR = 0.74,* p* = 0.32).

**Conclusion:**

Baseline SMI and PNI provide complementary prognostic information in patients with MBC receiving eribulin. Furthermore, on-treatment SMI gain, but not PNI increase, was associated with improved survival. Monitoring and enhancing skeletal muscle mass may improve outcomes, highlighting the importance of integrating supportive care strategies during chemotherapy.

**Supplementary Information:**

The online version contains supplementary material available at 10.1007/s10549-025-07827-y.

## Background

Sarcopenia, characterized by the loss of skeletal muscle and decreased physical activity in older adults [1], is associated with poor survival outcomes in various solid malignancies [2–4]. Generally, breast cancer tends to occur in younger female patients compared to other major cancers such as lung, gastric, and colorectal cancers [5]. As skeletal muscle mass decreases with age [6], patients with breast cancer typically have a higher skeletal muscle mass than those with other cancers. Consequently, the impact of skeletal muscle volume in patients with breast cancer may differ from that in patients with other cancer types. However, even in breast cancer, pretreatment with reduced skeletal muscle mass has been identified as a prognostic factor, particularly in patients with early breast cancer (EBC) [7–9].

Prognostic factors for patients with solid malignancies now encompass not only biological tumor characteristics, such as tumor size, breast cancer subtype, and gene expression in the primary tumor, but also host-related factors, such as skeletal muscle mass, nutritional status, and immunological status [10–15]. Increasing evidence suggests that these host factors have significant prognostic implications not only at baseline but also during their dynamic changes during cancer treatment [16, 17]. Previously, we reported that a decrease in the prognostic nutritional index (PNI), a systemic nutritional parameter calculated using serum albumin levels and lymphocyte counts [18], during neoadjuvant chemotherapy (NAC) is associated with worse survival in patients with EBC [15]. Similarly, a decrease in the skeletal muscle index (SMI), calculated as the standardized skeletal muscle area (SMA) at the third lumbar vertebral (L3) level divided by the square of the height [19], during NAC correlated with poor prognosis in patients with breast cancer [10]. These findings underscore the potential prognostic significance of dynamic changes in patient status during treatment. However, in contrast to EBC, the role of SMI, both at baseline and its dynamic changes during treatment, in metastatic breast cancer (MBC) remains unclear.

Eribulin mesylate (eribulin), a microtubule dynamics inhibitor, is widely used to treat MBC [20]. We previously identified the pretreatment PNI as a prognostic marker for patients with MBC receiving eribulin treatment [14]. Moreover, Miyoshi et al*.* reported that a high absolute lymphocyte count (ALC), an indicator of systemic immunological status [21], predicts better overall survival (OS) in patients with MBC undergoing eribulin treatment [12]. Hence, baseline nutritional and immunological status, represented by PNI and ALC, may be associated with survival outcomes in patients with MBC treated with eribulin. In contrast, although SMI is a widely recognized host-related prognostic factor, along with nutritional and immunological indicators, its association with prognosis after eribulin treatment in patients with MBC has not been reported. Moreover, the relationship between SMI and nutritional or immunological status in this population remains unclear.

In this study, we investigated the association between baseline SMI, changes in SMI during eribulin treatment, and the prognosis of patients with MBC. To this end, we measured SMI before treatment and at disease progression in patients with MBC treated with eribulin. By analyzing the impact of baseline SMI and SMI changes on patient outcomes, we aimed to clarify the prognostic significance of these factors in MBC. Furthermore, we investigated the correlation between SMI and PNI, and compared their differential prognostic impacts in this clinical context.

## Methods

### Patients and study design

In this retrospective study, we evaluated patients with MBC and an Eastern Cooperative Oncology Group performance status (PS) [22] of 0 or 1 who received eribulin treatment at Shinshu University Hospital between 2011 and 2022. The diagnosis of MBC was confirmed using radiographic imaging techniques, including computed tomography (CT) and/or ^18^F-fluorodeoxyglucose positron emission tomography (^18^F-FDG-PET/CT). Patients lacking comprehensive clinical data were excluded, resulting in a cohort of 67 patients (Supplementary Fig. 1). This study conformed to the provisions of the Declaration of Helsinki (64th WMA General Assembly, Fortaleza, Brazil, October 2013). This study was approved by the local ethics committee on the clinical investigation of Shinshu University (no. 4672). Given the retrospective design with anonymized data, the need for informed consent was waived. Our institution uses a form on its website to enable patients to opt out of the use of their clinical data for research purposes.

### Eribulin treatment protocol

Eribulin was administered intravenously at 1.4 mg/m^2^ on days 1 and 8 of each 21-day cycle. In cases of grade 3 or 4 hematological toxicity or febrile neutropenia, as defined by the Common Terminology Criteria for Adverse Events (CTCAE) version 5.0, day 8 treatment was skipped until day 15. Additionally, the dose was reduced stepwise to 1.1 mg/m^2^ or 0.7 mg/m^2^ as necessary.

### Data collection

Clinical data were retrospectively collected from medical records and included age at the initiation of eribulin treatment, menopausal status, PS, estrogen receptor (ER) status, progesterone receptor (PgR) and human epidermal growth factor receptor type 2 (HER2) status, previous treatments, number of chemotherapy regimens before eribulin treatment, and metastatic sites. Neoadjuvant or adjuvant chemotherapy with anthracycline and/or taxane-based regimens was excluded from prior chemotherapy regimens. Metastatic sites were categorized as either visceral (lung, liver, and brain) or non-visceral (locoregional soft tissues, skin, and bone). Tumor response to eribulin treatment was assessed according to the RECIST version 1.1 [23] before and at week 6 (± 3 weeks) after the initial eribulin administration. Progression-free survival (PFS) was defined as the period from the first day of eribulin administration to treatment cessation due to disease progression. OS was calculated from the first eribulin administration until death from any cause.

### Calculation of skeletal muscle mass index and prognostic nutritional index

SMA was measured using CT or ^18^F-FDG-PET/CT before the start of eribulin treatment. ^18^F-FDG PET/CT scans were performed using a Discovery ST Elite Performance Scanner (GE Healthcare, Tokyo, Japan). Attenuation-corrected images were reconstructed in the coronal plane. SMA was quantified by semi-automatic tracing of muscles (including the psoas, paraspinal, transversus abdominis, rectus abdominis, and internal and external obliques) at L3, visualized within a range of − 29 to 150 Hounsfield units using the EV Insite R (PSP Corporation, Tokyo, Japan) system and expressed in cm^2^ as previously described [10, 24]. SMI was calculated by dividing the SMA by height squared (m^2^) (Supplementary Fig. 2) [25, 26]. The percent change in SMI was calculated as the percent change in SMI at disease progression from the pretreatment baseline.

PNI values were calculated from the results of routine blood examination performed several days before or on the day of first administration of eribulin using the following formula: 10 × serum albumin value (g/dl) + 0.005 × total lymphocyte count in the peripheral blood/mm^3^ [18]. The change in PNI was calculated as each value at pretreatment minus that at disease progression.

### Statistical analyses

Categorical variables were analyzed using the chi-squared test or Fisher’s exact test, and continuous variables were assessed using the Mann–Whitney *U* test. Survival curves were estimated using the Kaplan–Meier method, with significant differences in survival determined using the log-rank test. All statistical analyses were performed using GraphPad Prism 10.2.3 (GraphPad Software, CA, USA), and *p* < 0.05 was considered statistically significant.

## Results

### Baseline patient characteristics

The clinicopathological characteristics of the 67 patients are summarized in Table 1. The mean age (± standard deviation) was 58.9 ± 12.0 years. Among them, 19 (28.4%) were premenopausal and 48 (71.6%) were postmenopausal. The mean pretreatment PNI (pre-PNI) was 45.5 ± 6.1. Regarding subtype, 54 patients (80.6%) had ER-positive breast cancer, 45 patients (67.2%) had PgR-positive breast cancer, and 57 patients (85.1%) had HER2-negative breast cancer. Sixty-two patients (91.5%) had previously received anthracycline- and/or taxane-based chemotherapy in neoadjuvant, adjuvant, or metastatic settings [both agents: *n* = 51 (76.1%); anthracycline only: *n* = 3 (4.5%); taxane only: *n* = 8 (11.9%)]. Before initiating eribulin treatment, 48 patients received other chemotherapy regimens, including chemotherapy alone (*n* = 42, 62.7%) and chemotherapy combined with anti-HER2 therapy (n = 6, 9.0%). Fourteen patients (20.9%) received endocrine therapy before eribulin treatment. Twenty-one patients (31.3%) underwent either one chemotherapeutic regimen or none, whereas the remaining 46 patients (68.7%) received at least two regimens. Visceral metastases were observed in 57 patients (85.1%). Regarding PS, 46 patients (68.7%) had a PS score of 0, and 21 patients (31.3%) had a PS of 1. In terms of clinical response, 26 patients (38.8%) showed partial response (PR), 17 (25.4%) exhibited stable disease (SD)/long SD, and 24 (35.8%) had progressive disease (PD). The median follow-up period was 13.8 months (range = 1.6–85.3 months).

**Table 1:** Patients’ clinical features classified by pre-SMI levels

Variables	Total (%)	Pre-SMI		*p* value
		High (%)	Low (%)	
	n = 67 (100)	n = 28 (41.8)	n = 39 (58.2)	
Age (y.o. mean ± SD)	58.9 ± 12.0	61.8 ± 11.2	56.9 ± 12.1	0.10
Menopausal status				
Premenopausal	19 (28.4)	6 (21.4)	13 (33.3)	0.41
Postmenopausal	48 (71.6)	22 (78.6)	26 (66.7)	
PNI (mean ± SD)	45.5 ± 6.1	47.1 ± 5.6	44.3 ± 6.4	0.08
ER				
Positive	54 (80.6)	25 (89.3)	29 (74.4)	0.21
Negative	13 (19.4)	3 (10.7)	10 (25.6)	
PgR				
Positive	45 (67.2)	20 (71.4)	25 (64.1)	0.60
Negative	22 (32.8)	8 (28.6)	14 (35.9)	
HER2				
Positive	10 (14.9)	4 (14.3)	6 (15.4)	0.99
Negative	57 (85.1)	24 (85.7)	33 (84.6)	
Previous anthracycline and taxane-based therapy				
Both	51 (76.1)	21 (75.0)	30 (76.9)	0.16
Anthracycline only	3 (4.5)	3 (10.7)	0 (0)	
Taxane only	8 (11.9)	3 (10.7)	5 (12.8)	
None	5 (7.5)	1 (3.6)	4 (10.3)	
Treatment type before eribulin				
Chemotherapy	42 (62.7)	19 (67.9)	23 (59.0)	0.75
Chemotherapy and anti-HER2 therapy	6 (9.0)	1 (3.6)	5 (12.8)	
Anti-HER2 therapy	3 (4.5)	1 (3.6)	2 (5.1)	
Endocrine therapy	14 (20.9)	6 (21.4)	8 (20.5)	
None	3 (4.5)	1 (3.6)	2 (5.1)	
Number of chemotherapy regimens prior to eribulin				
0—1	21 (31.3)	9 (32.1)	12 (30.8)	> 0.99
≥ 2	46 (68.7)	19 (67.9)	27 (69.2)	
Metastatic site				
Visceral	57 (85.1)	22 (78.6)	35 (89.7)	0.30
Non-visceral	10 (14.9)	6 (21.4)	4 (10.3)	
Performance status				
0	46 (68.7)	19 (67.9)	27 (69.2)	0.99
1	21 (31.3)	9 (32.1)	12 (30.8)	
Clinical response				
PR	26 (38.8)	12 (42.9)	14 (35.9)	0.58
SD or long SD	17 (25.4)	8 (28.6)	9 (23.1)	
PD	24 (35.8)	8 (28.6)	16 (41.0)	

*PNI* prognostic nutritional index, *SMI* skeletal muscle index, *ER* estrogen receptor, *PgR* progesterone receptor, *HER2* human epidermal growth factor receptor type2, *PR* partial response, *SD* stable disease, *PD* progressive disease

### Association between pretreatment skeletal muscle index (SMI) and prognosis

**Fig. 1:**
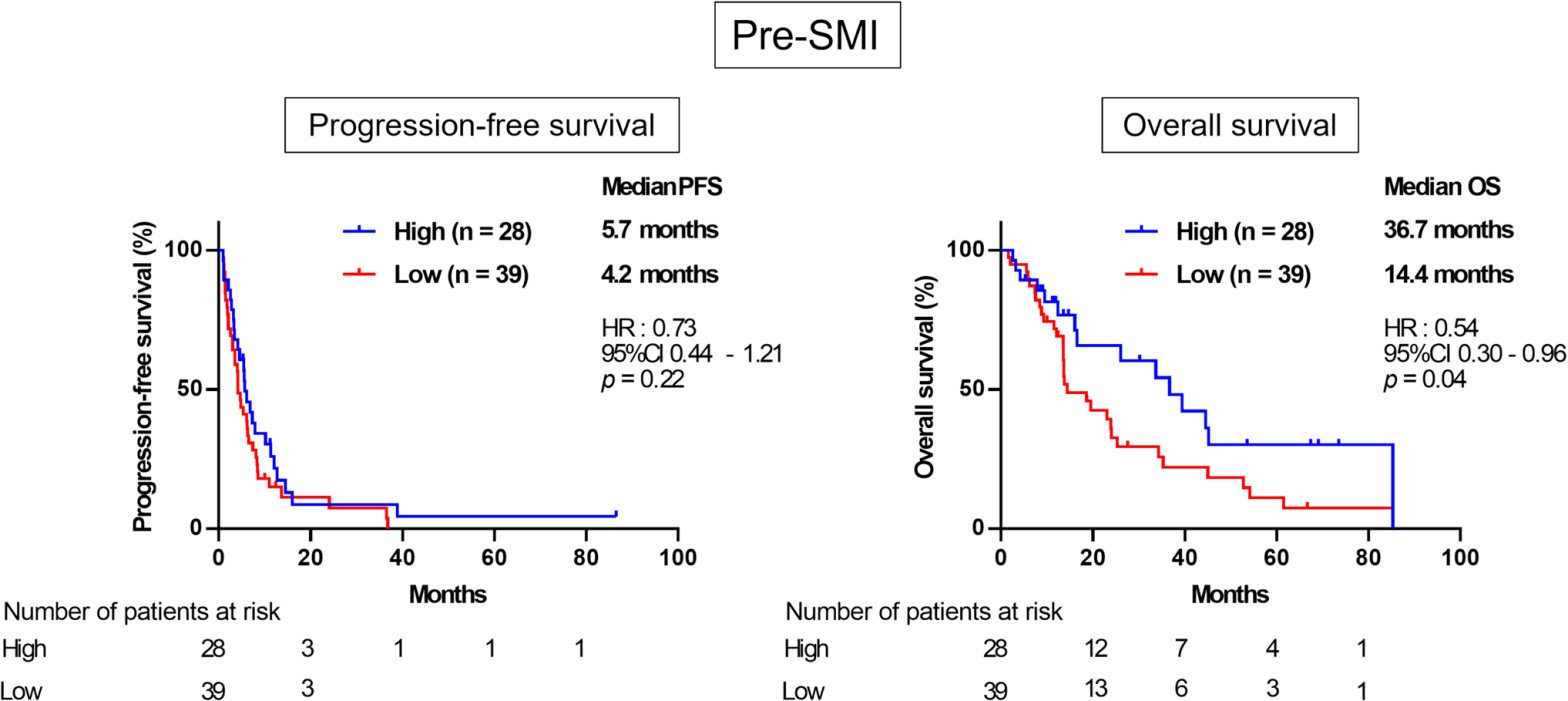
Kaplan–Meier curves for PFS (left) and OS (right) according to baseline SMI (Pre-SMI). PFS, HR 0.73; 95%CI 0.44–1.21; *p* = 0.22. OS, HR 0.54; 95%CI 0.30–0.96; *p* = 0.04. *PFS* progression-free survival, *OS* overall survival, *SMI* skeletal muscle index, *HR* hazard ratio, *CI* confidence interval

The mean pretreatment SMI (pre-SMI) was 42.8 ± 8.7. To evaluate the correlation between pre-SMI and prognosis after eribulin treatment, we classified the patients into high and low pre-SMI groups according to the cutoff value for OS (43.3, area under the curve = 0.66), which was determined by receiver operating characteristic (ROC) curve analysis, and compared the PFS and OS in the high and low pre-SMI groups. There were no significant differences in clinicopathological features between the high and low pre-SMI groups (Table 1). Although there was no significant difference in PFS between the pre-SMI high and low groups [hazard ratio (HR) = 0.73; 95% confidence interval (CI) = 0.45–1.21; *p* = 0.27], the pre-SMI high group showed significantly better OS compared to the pre-SMI low group [HR = 0.54; 95% CI = 0.30–0.96; *p* = 0.04] (Fig. 1).

### Association between pretreatment SMI and changes in SMI during eribulin therapy

**Fig. 2:**
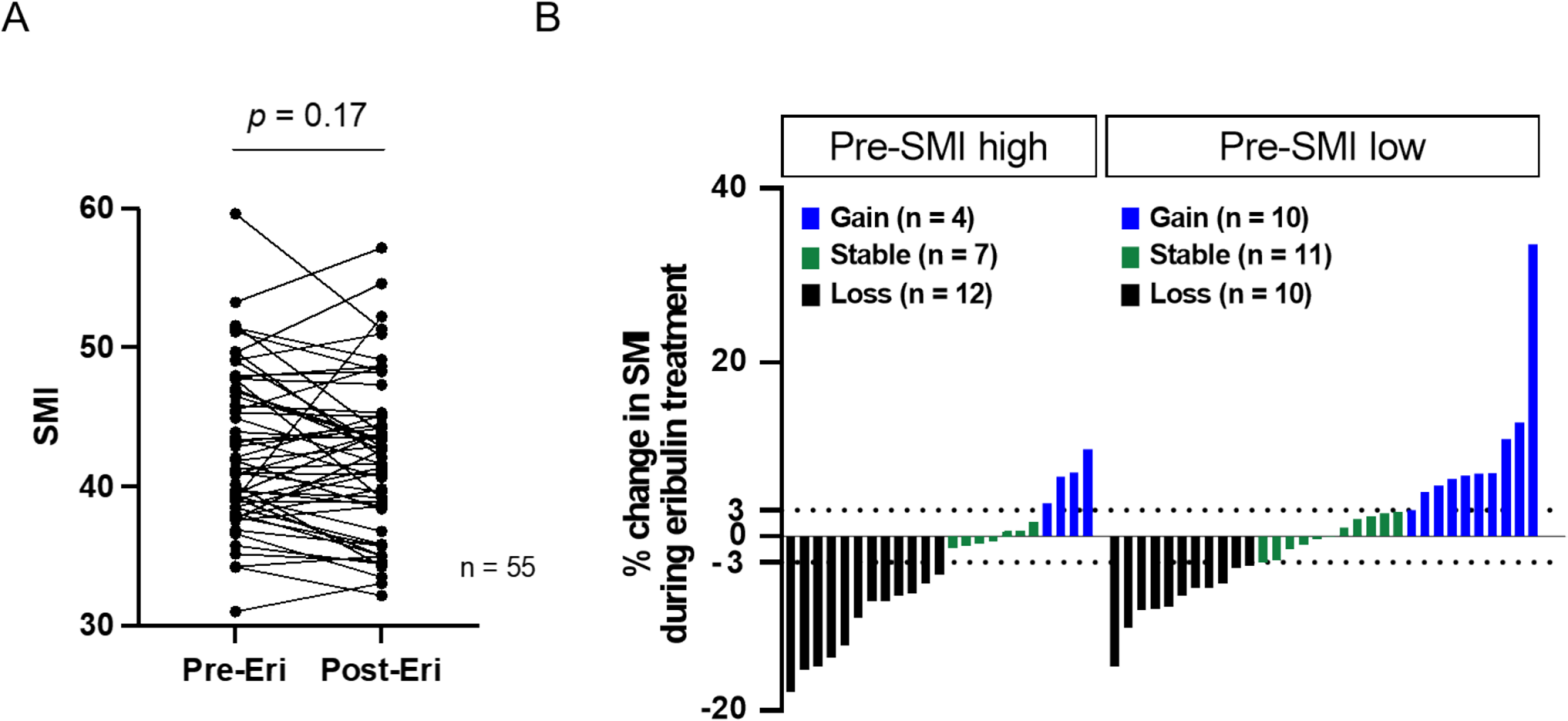
**A** Baseline SMI (Pre-Eri) and SMI at disease progression (Post-Eri) of individual patient (*p* = 0.17). **B** Percent Change in SMI during eribulin treatment of individual patient in the Pre-SMI high (left) and the pre-SMI low group (right). Gain was defined as more than 3% increase, Stable as within −3% to 3% change, and loss as more than 3% decrease, respectively. *SMI* skeletal muscle index, *Eri* eribulin

Next, we focused on changes in SMI during eribulin treatment and their association with patient outcomes. Post-treatment SMI (post-SMI) was measured at the time of disease progression. Among the 67 patients, post-SMI data were available for 55. In these 55 patients, there was no significant difference between the pre- and post-SMI values (*p* = 0.17) (Fig. 2A). Based on changes in SMI before and after eribulin treatment, patients were categorized into three groups: gain (≥ 3% increase), stable (between − 3% and  < 3% change), and loss (a ≥  − 3% decrease) during eribulin treatment, as previously described [10]. In the pre-SMI high group, 4 (17.4%), 7 (30.4%), and 12 (52.2%) patients were in the gain, stable, and loss groups, respectively. In the pre-SMI low group, 10 (31.2%), 11 (34.4%), and 11 (34.4%) patients were assigned to the gain, stable, and loss groups, respectively (Fig. 2B). This distribution of SMI change showed no significant difference between the high and low pre-SMI groups (*p* = 0.40), indicating that the change in SMI was not dependent on pre-SMI.

### Association between changes in SMI during eribulin therapy and prognosis

**Fig. 3:**
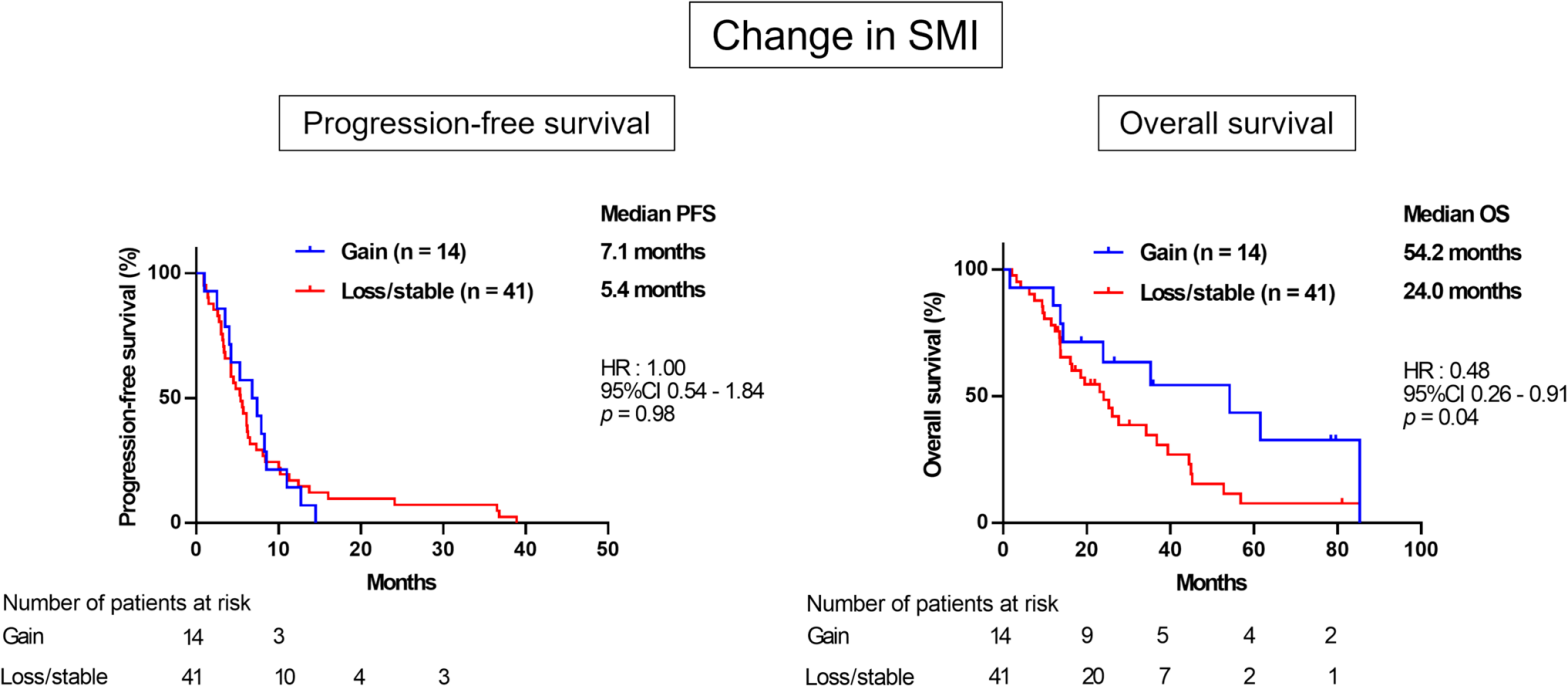
Kaplan–Meier curves for PFS (left) and OS (right) according to change in SMI. DFS, HR 1.00; 95%CI 0.54–1.84; *p* = 0.98. OS, HR 0.48; 95%CI 0.26–0.91; *p* = 0.04. *PFS* progression-free survival, *OS* overall survival, *SMI* skeletal muscle index, *HR* hazard ratio, *CI* confidence interval

We then analyzed the association between patient outcomes and changes in SMI during eribulin treatment. Patients were categorized into two either the gain group or the combined loss/stable group, and their clinicopathological characteristics and prognoses were compared. There were no significant differences in any of the parameters between the gain group and the loss/stable group (Table 2). While PFS did not differ significantly between two groups [HR = 1.00; 95% CI = 0.54–1.84; *p* = 0.98], OS was significantly longer in the gain group than the loss/stable group [HR = 0.48; 95% CI = 0.26–0.91; *p* = 0.04] (Fig. 3).

**Table 2:** Comparison of characteristics of the patients between the SMI gain and the SMI loss/stable group

Variables	SMI		*p* value
	Gain (%)	Loss/stable (%)	
	14 (25.5)	41 (74.5)	
Age (y.o. mean ± SD)	57.6 ± 9.5	59.6 ± 12.6	0.59
Menopausal status			
Premenopausal	4 (28.6)	10 (24.1)	0.74
Postmenopausal	10 (71.4)	31 (75.6)	
PNI (mean ± SD)	46.8 ± 6.5	45.7 ± 5.8	0.59
ER			
Positive	11 (78.6)	33 (80.5)	> 0.99
Negative	3 (21.4)	8 (19.5)	
PgR			
Positive	10 (71.4)	27 (65.9)	> 0.99
Negative	4 (28.6)	14 (34.1)	
HER2			
Positive	2 (14.3)	5 (12.2)	> 0.99
Negative	12 (85.7)	36 (87.8)	
Previous anthracycline and taxane-based therapy			
Both	11 (78.6)	29 (70.7)	0.57
Anthracycline only	1 (7.1)	2 (4.9)	
Taxane only	0 (0)	7 (17.1)	
None	2 (14.3)	3 (7.3)	
Treatment type before eribulin			
Chemotherapy	11 (78.6)	23 (56.1)	0.12
Chemotherapy and anti-HER2 therapy	2 (14.3)	1 (2.4)	
Anti-HER2 therapy	0 (0)	2 (4.9)	
Endocrine therapy	1 (7.1)	13 (31.7)	
None	0 (0)	2 (4.9)	
Number of chemotherapy regimens prior to eribulin			
0—1	3 (21.4)	15 (36.6)	0.21
≥ 2	11 (78.6)	26 (63.4)	
Metastatic site			
Visceral	13 (92.9)	35 (85.4)	0.66
Non-visceral	1 (7.1)	6 (14.6)	
Performance status			
0	12 (85.7)	27 (65.9)	0.19
1	2 (14.3)	14 (34.1)	

*PNI* prognostic nutritional index, *SMI* skeletal muscle index, *ER* estrogen receptor, *PgR* progesterone receptor, *HER2* human epidermal growth factor receptor type2

One possible explanation for the poorer prognosis in the loss/stable group is that these patients might have been less able to receive subsequent chemotherapy owing to physical decline. To investigate this possibility, we compared the number of chemotherapy regimens administered after eribulin between the two groups (Table 3). The number of subsequent regimens did not differ significantly between the gain and loss/stable groups (*p* = 0.75), suggesting that differences in post-eribulin treatment exposure did not account for the OS disparity.

**Table 3:** Comparison of treatment after eribulin between the SMI gain and the SMI loss/stable group

	SMI		*p* value
	Gain (%)	Loss/stable (%)	
Number of chemotherapy regimens after eribulin	n = 14	*n* = 41	
0–1	5 (35.7)	18 (43.9)	0.75
≥ 2	9 (64.3)	23 (56.1)	

SMI: skeletal muscle index

### Association between SMI and PNI

**Fig. 4:**
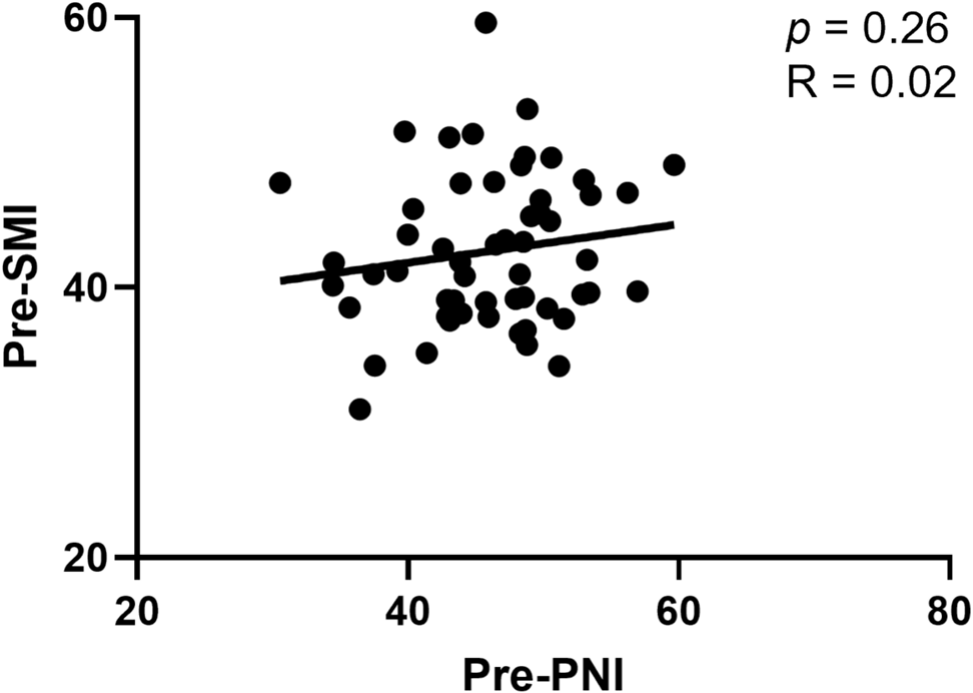
Scatter plot of pretreatment SMI (Pre-SMI) against pretreatment PNI (Pre-PNI). Correlation is shown using Pearson correlation coefficients (R) (R = 0.02), and significance was determined using the Spearman correlation (*p* = 0.026). *SMI* skeletal muscle index, *PNI* prognostic nutritional index

We previously demonstrated that pretreatment PNI is a prognostic factor on eribulin treatment in patients with MBC [14]. Although PNI and SMI are both host-related factors, their correlation in patients with MBC remains unclear. Therefore, we examined the relationship between SMI and PNI. The correlation between pre-SMI and pre-PNI was negligible (*p* = 0.26, *R* = 0.02), indicating no meaningful linear association (Fig. 4).

Next, we assessed the relationship between changes in SMI and PNI during eribulin therapy. Among 55 patients with available post-SMI data, PNI increased and decreased in 20 and 35, respectively (Supplementary Fig. 3). The distribution of changes in SMI and PNI is presented in Supplementary Table 1. Among 14 patients with SMI gain, PNI increased in seven (50.0%) and decreased in seven (50.0%). Meanwhile, among 41 patients with SMI loss or stability, PNI increased and decreased in 13 (31.7%) and 28 (68.3%), respectively. This difference was not statistically significant (*p* = 0.33), suggesting that SMI and PNI are independent factors.

### Prognostic impact of pretreatment PNI and on-treatment PNI change

**Fig. 5:**
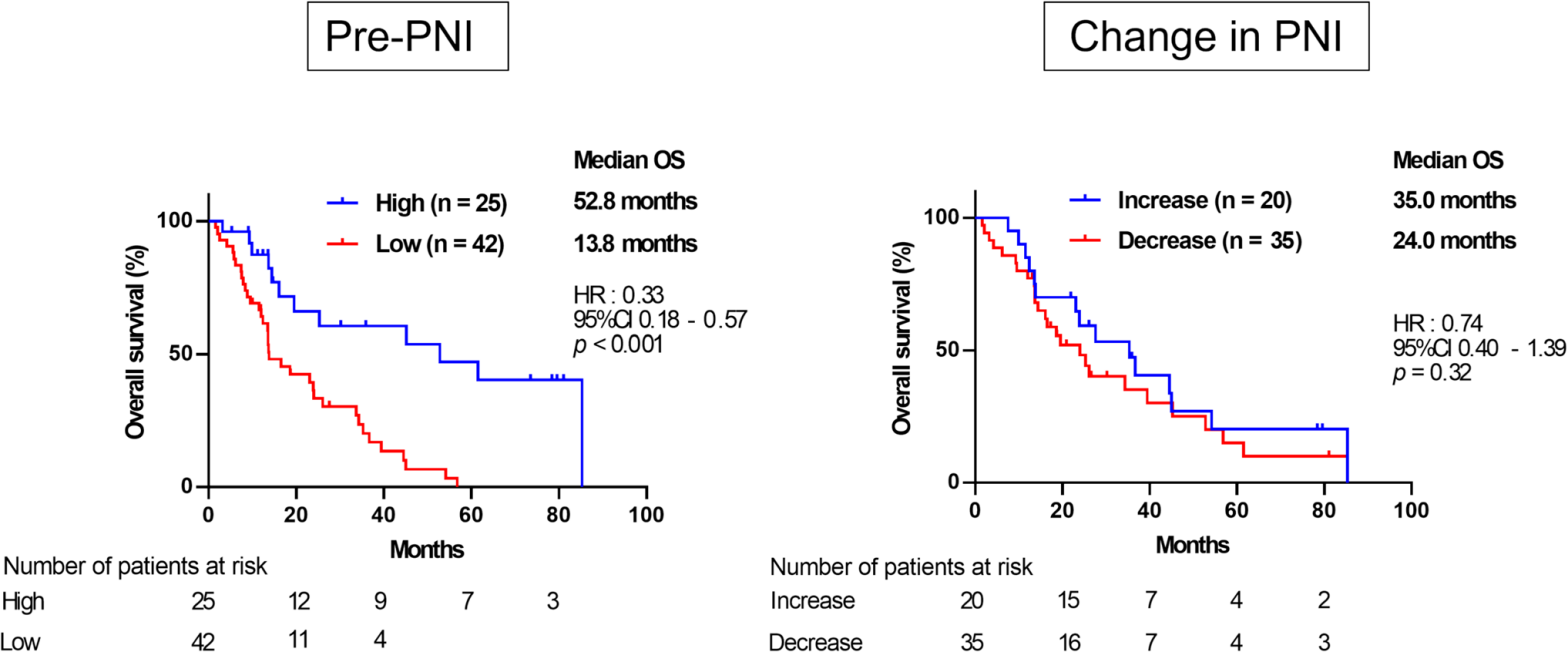
Kaplan–Meier curves for OS according to pretreatment PNI (HR = 0.33; 95% CI = 0.18–0.57; *p* < 0.001) (left) and change in PNI (HR = 0.74; 95% CI = 0.40–1.39; *p* = 0.32) (right). *OS* overall survival, *PNI* prognostic nutritional index, *HR* hazard ratio, *CI* confidence interval

Finally, we evaluated the prognostic impact of PNI. Patients were stratified into high and low pre-PNI groups using a cutoff value of 48.3, as previously described [14]. The high pre-PNI group had significantly longer OS than the low pre-PNI group [HR = 0.33; 95% CI = 0.18–0.57; *p* < 0.001] (Fig. 5a). Subsequently, we analyzed the prognostic impact of on-treatment PNI changes by categorizing patients into increase and decrease groups. OS did not differ significantly between the two groups [HR = 0.74; 95% CI = 0.40–1.39; *p* = 0.32] (Fig. 5b). These findings indicate that pretreatment PNI is a prognostic marker in patients with MBC treated with eribulin, whereas changes in PNI during treatment do not have a significant prognostic effect.

## Discussion

This study demonstrated that patients with a high pre-SMI exhibited better prognosis after eribulin treatment than those with a low pre-SMI. Additionally, an increase in SMI during eribulin treatment was significantly associated with favorable outcomes.

Accumulating evidence suggests that tumor biology and host-related factors affect patient outcomes in various solid malignancies [10–15]. Kashiwagi et al. previously demonstrated that a high density of tumor-infiltrating lymphocytes (TILs) in the tumor microenvironment (TME) was associated with a better prognosis in patients with triple-negative breast cancer receiving eribulin treatment [11]. This suggests that a robust local immune response in the TME correlates with improved patient prognosis. Furthermore, local immune conditions in the TME and systemic immune parameters such as the PNI and ALC are associated with survival outcomes in various solid malignancies [27]. These findings suggest that host immunological status can be a representative host-related factor with prognostic significance in eribulin treatment. Another important host-related factor is skeletal muscle mass, which has been associated with prognosis in patients with several types of solid tumors, including breast cancer [25, 28, 29]. However, its effect on patients with MBC undergoing eribulin treatment remains unexplored. Our study addresses this gap by demonstrating that skeletal muscle mass at the start of eribulin treatment may serve as a prognostic factor for patient outcomes in MBC.

While the prognostic value of pre-SMI is well recognized in cancer treatment, more attention has recently been paid to dynamic changes in skeletal muscle mass during chemotherapy in individual patients. Emerging evidence suggests that loss of skeletal muscle mass is associated with worse outcomes in patients with solid tumors, including gastric and other cancers [30, 31]. Our previous study demonstrated that a decrease in SMI was associated with poor prognosis in patients with EBC [10]. Typically, patients with MBC are more likely to develop sarcopenia than those with EBC. This is, at least partly, due to prior chemotherapy in patients with MBC, which can reduce food intake because of gastrointestinal side effects and decrease physical activity due to general fatigue [32]. Additionally, cachexia resulting from tumor progression can contribute to a decline in SMI [33]. Hence, the impact of changes in SMI may differ between patients with EBC and those with MBC. Our study helps fill this knowledge gap by demonstrating that changes in skeletal muscle mass in patients with MBC also have prognostic significance.

Although both SMI and PNI are host-related prognostic factors, they represent distinct biological aspects: SMI reflects body composition, whereas PNI is derived from nutritional and immunological laboratory parameters. While weak positive correlations between these indices have been reported in gastrointestinal cancers [34, 35], their relationship in breast cancer has been underexplored. In this study, no significant correlation was observed between SMI and PNI. Importantly, both baseline SMI and on-treatment SMI change affected OS, whereas only baseline PNI, but not on-treatment PNI change, was prognostic. These findings suggest that SMI and PNI independently influence outcomes and that skeletal muscle mass represents a more dynamic prognostic marker during therapy. Overall, improving nutritional status before eribulin initiation and maintaining or enhancing skeletal muscle mass during treatment may contribute to improved survival.

In this study, although PFS was not significantly longer in either the pre-SMI high group or the SMI-gain group compared to the pre-SMI low group or the SMI-stable/loss group, OS was significantly longer in both groups. This suggests that SMI is not a predictive factor for response to eribulin treatment but is a prognostic factor from the initiation of treatment. The observed improvement in OS may be attributed to sustained tolerance to subsequent chemotherapy after eribulin treatment in patients with a high pre-SMI or an increase in SMI during eribulin treatment. However, in our study, the number of post-eribulin chemotherapy regimens did not differ between the gain and loss/stable groups. Notably, eribulin itself is known to prolong OS without significantly improving PFS in patients with MBC compared to treatment at the physician’s choice [20]. Several mechanisms have been proposed to explain this OS benefit, including remodeling of abnormal tumor vasculature [36], induction of mesenchymal-epithelial transition in cancer cells [37], and facilitation of CD8^+^ T cell proliferation [38], highlighting the multifaceted effects of eribulin on various cell types. Through these immunomodulatory effects on the TME, eribulin may improve the efficacy of subsequent chemotherapy administered after eribulin. Together with the findings of this study that OS benefit might be stronger in patients with SMI gain than in those with SMI stable/loss, the immunomodulatory effects of eribulin on the TME are more pronounced in patients with increased in SMI during treatment. Furthermore, the present study revealed that approximately one-quarter of patients experienced an increase in SMI during eribulin treatment. This observation contrasts with the common trend of SMI reduction during chemotherapy, which is often caused by treatment-induced malnutrition and reduced physical activity [32]. When considered alongside the aforementioned effects of eribulin, this finding suggests that eribulin may exert direct or indirect effects on skeletal muscle cells, potentially contributing to increased skeletal muscle mass. However, it is important to note that this study exclusively examined patients treated with eribulin, excluding those who received other chemotherapeutic agents. Further research encompassing patients treated with a broader range of chemotherapy options is necessary to validate this hypothesis and to fully elucidate the mechanisms of eribulin and the role of SMI in survival outcomes.

The findings of this study highlight the importance of monitoring and maintaining skeletal muscle mass during chemotherapy for MBC. Appropriate supportive care, including nutritional interventions and physical activity, aimed at increasing skeletal muscle mass during chemotherapy could improve the prognosis of patients with breast cancer. Particularly, physical activity has been reported to reduce the risk of developing breast cancer [39], and engaging in physical activity after a breast cancer diagnosis has been associated with reduced mortality rates [40]. A recent study demonstrated that moderate physical activity during NAC in patients with breast cancer was correlated with a higher rate of pathological complete response, suggesting that physical activity may improve treatment outcomes [41]. Additionally, a recent randomized controlled trial showed that nutritional interventions in patients with MBC improved obesity and reduced serum cholesterol levels. Although this study did not assess the impact on prognosis, it suggests that such interventions may contribute to improvements in overall health in patients with MBC [42]. Several clinical trials are currently ongoing to evaluate whether combining physical activity and nutritional support with standard therapy can improve outcomes in patients with cancer. These include a phase III trial (NCT02330926) for lung cancer and a phase II trial (UMIN000028801) for lung cancer and pancreatic cancer. The results are eagerly awaited.

This study has several limitations. First, it was a retrospective analysis conducted at a single institution, which may limit the generalizability of the findings to other populations. Second, the sample size was relatively small, potentially reducing the statistical power to detect significant associations. Third, post-SMI was measured at the time of disease progression, and the timing of this evaluation varied across patients, potentially introducing variability in the assessment of SMI changes. Lastly, the study focused solely on patients treated with eribulin without comparison with other chemotherapeutic agents, making it unclear whether the observed associations between SMI and prognosis are specific to eribulin or applicable to other treatments. Further studies with larger cohorts and a more standardized timing of SMI evaluations are warranted to validate our findings.

## Conclusion

In patients with MBC receiving eribulin, both higher baseline SMI and on-treatment SMI gain were associated with improved OS, whereas PNI was prognostic only at baseline. Our results highlight the importance of improving pretreatment nutritional status and skeletal muscle mass and increasing skeletal muscle mass during chemotherapy for MBC.

## Supplementary Information

Below is the link to the electronic supplementary material.

Supplementary file1 (DOCX 39 KB)

Supplementary file2 (PPTX 41 KB)

Supplementary file3 (PPTX 364 KB)

Supplementary file4 (PPTX 59 KB)

Supplementary file5 (DOCX 16 KB)

## Data Availability

The data supporting the findings of this work are available from the authors upon reasonable request.

## Abbreviations

SMI: Skeletal muscle index
MBC: Metastatic breast cancer
EBC: Early breast cancer
PNI: Prognostic nutritional index
NAC: Neoadjuvant chemotherapy
SMA: Skeletal muscle area
ALC: Absolute lymphocyte count
ER: Estrogen receptor
PgR: Progesterone receptor
HER2: Human epidermal growth factor receptor type 2
SD: Stable disease
PD: Progressive disease
TILs: Tumor-infiltrating lymphocytes
TME: Tumor microenvironment

## References

[1] Cruz-Jentoft AJ, Bahat G, Bauer J, Boirie Y, Bruyere O, Cederholm T, Cooper C, Landi F, Rolland Y, Sayer AA, Schneider SM, Sieber CC, Topinkova E, Vandewoude M, Visser M, Zamboni M, Writing Group for the European Working Group on Sarcopenia in Older P, the Extended Group for E (2019) Sarcopenia: revised European consensus on definition and diagnosis. Age Ageing 48:16–31 30312372 10.1093/ageing/afy169 PMC6322506

[2] Shachar SS, Williams GR, Muss HB, Nishijima TF (2016) Prognostic value of sarcopenia in adults with solid tumours: a meta-analysis and systematic review. Eur J Cancer 57:58–67 26882087 10.1016/j.ejca.2015.12.030

[3] Daly LE, ÉB NB, Power DG, Cushen SJ, James K, Ryan AM (2018) Loss of skeletal muscle during systemic chemotherapy is prognostic of poor survival in patients with foregut cancer. J Cachexia Sarcopenia Muscle 9:315–325 29318756 10.1002/jcsm.12267 PMC5879982

[4] Park SE, Choi JH, Park JY, Kim BJ, Kim JG, Kim JW, Park JM, Chi KC, Hwang IG (2020) Loss of skeletal muscle mass during palliative chemotherapy is a poor prognostic factor in patients with advanced gastric cancer. Sci Rep 10:17683 33077864 10.1038/s41598-020-74765-8 PMC7573603

[5] Siegel RL, Giaquinto AN, Jemal A (2024) Cancer statistics, 2024. CA Cancer J Clin 74:12–49 38230766 10.3322/caac.21820

[6] Janssen I, Heymsfeld SB, Wang Z, Robert R (2000) Skeletal muscle mass and distribution in 468 men and women aged 18–88 yr. J Appl Physiol 89:81–88 10904038 10.1152/jappl.2000.89.1.81

[7] Zhang XM, Dou QL, Zeng Y, Yang Y, Cheng ASK, Zhang WW (2020) Sarcopenia as a predictor of mortality in women with breast cancer: a meta-analysis and systematic review. BMC Cancer 20:172 32131764 10.1186/s12885-020-6645-6 PMC7057618

[8] Roberto M, Barchiesi G, Resuli B, Verrico M, Speranza I, Cristofani L, Pediconi F, Tomao F, Botticelli A, Santini D (2024) Sarcopenia in breast cancer patients: a systematic review and meta-analysis. Cancers (Basel). 10.3390/cancers16030596 38339347 10.3390/cancers16030596 PMC10854936

[9] Aktas A, Greiner RS, Flores M, Boselli D, Stone T, Wang E, Hadzikadic-Gusic L, Wallander ML, Hecksher A, Bailey-Dorton C, Walsh D (2025) Association of skeletal muscle mass and muscle quality at diagnosis with survival in young women with breast cancer: retrospective observational study. Clin Breast Cancer 25:223–232 39578152 10.1016/j.clbc.2024.10.014

[10] Amitani M, Oba T, Kiyosawa N, Morikawa H, Chino T, Soma A, Shimizu T, Ohno K, Ono M, Ito T, Kanai T, Maeno K, Ito KI (2022) Skeletal muscle loss during neoadjuvant chemotherapy predicts poor prognosis in patients with breast cancer. BMC Cancer 22:327 35346102 10.1186/s12885-022-09443-1 PMC8962250

[11] Kashiwagi S, Asano Y, Goto W, Takada K, Takahashi K, Noda S, Takashima T, Onoda N, Tomita S, Ohsawa M, Hirakawa K, Ohira M (2017) Use of tumor-infiltrating lymphocytes (TILs) to predict the treatment response to eribulin chemotherapy in breast cancer. PLoS ONE 12:e0170634 28166544 10.1371/journal.pone.0170634 PMC5293550

[12] Miyoshi Y, Yoshimura Y, Saito K, Muramoto K, Sugawara M, Alexis K, Nomoto K, Nakamura S, Saeki T, Watanabe J, Perez-Garcia JM, Cortes J (2020) High absolute lymphocyte counts are associated with longer overall survival in patients with metastatic breast cancer treated with eribulin-but not with treatment of physician’s choice-in the EMBRACE study. Breast Cancer 27:706–715 32133606 10.1007/s12282-020-01067-2 PMC7297864

[13] Oba T, Maeno K, Amitani M, Shimizu T, Ohno K, Ono M, Ito T, Kanai T, Uehara T, Ito KI (2021) Prognostic significance of neutrophil-to-lymphocyte ratio for long-term outcomes in patients with poorly differentiated thyroid cancer. Endocr J. 10.1507/endocrj.EJ21-0237 34219074 10.1507/endocrj.EJ21-0237

[14] Oba T, Maeno K, Ono M, Ito T, Kanai T, Ito KI (2021) Prognostic nutritional index is superior to neutrophil-to-lymphocyte ratio as a prognostic marker in metastatic breast cancer patients treated with Eribulin. Anticancer Res 41:445–452 33419842 10.21873/anticanres.14794

[15] Oba T, Maeno K, Takekoshi D, Ono M, Ito T, Kanai T, Ito KI (2020) Neoadjuvant chemotherapy-induced decrease of prognostic nutrition index predicts poor prognosis in patients with breast cancer. BMC Cancer 20:160 32106833 10.1186/s12885-020-6647-4 PMC7045374

[16] Kim J-Y, Jung EJ, Kim J-M, Lee HS, Kwag S-J, Park J-H, Park T, Jeong S-H, Jeong C-Y, Ju Y-T (2020) Dynamic changes of neutrophil-to-lymphocyte ratio and platelet-to-lymphocyte ratio predicts breast cancer prognosis. BMC Cancer. 10.1186/s12885-020-07700-9 33287745 10.1186/s12885-020-07700-9 PMC7720486

[17] Lin CH, Chou WC, Wu YY, Lin CY, Chang KP, Liao CT, Ho TY, Yeh CM, Liu CJ, Hung SP, Lee CH, Chen PJ, Chou YC, Fan KH, Huang BS, Tung-Chieh Chang J, Wang CC, Tsang NM (2021) Prognostic significance of dynamic changes in lymphocyte-to-monocyte ratio in patients with head and neck cancer treated with radiotherapy: results from a large cohort study. Radiother Oncol 154:76–86 32941957 10.1016/j.radonc.2020.09.012

[18] Onodera T, Goseki N, Kosaki G (1984) Prognostic nutritional index in gastrointestinal surgery of malnourished cancer patients. Nihon Geka Gakkai Zasshi 85:1001–1005 6438478

[19] Blauwhoff-Buskermolen S, Versteeg KS, de van der Schueren MA, den Braver NR, Berkhof J, Langius JA, Verheul HM (2016) Loss of muscle mass during chemotherapy is predictive for poor survival of patients with metastatic colorectal cancer. J Clin Oncol 34:1339–1344 26903572 10.1200/JCO.2015.63.6043

[20] Cortes J, O’ Shaughnessy J, Loesch D, Blum JL, Vahdat LT, Petrakova K, Chollet P, Manikas A, Dieras V, Delozier T, Vladimirov V, Cardoso F, Koh H, Bougnoux P, Dutcus CE, Seegobin S, Mir D, Meneses N, Wanders J, Twelves C, Investigators E (2011) Eribulin monotherapy versus treatment of physician’s choice in patients with metastatic breast cancer (EMBRACE): a phase 3 open-label randomised study. Lancet 377:914–923 21376385 10.1016/S0140-6736(11)60070-6

[21] Qian Y, Tao J, Li X, Chen H, Lu Q, Yang J, Pan H, Wang C, Zhou W, Liu X (2018) Peripheral inflammation/immune indicators of chemosensitivity and prognosis in breast cancer patients treated with neoadjuvant chemotherapy. OncoTargets Ther 11:1423–1432 10.2147/OTT.S148496 PMC5858818 29588597

[22] Oken MM, Creech RH, Tormey DC, Horton J, Davis TE, McFadden ET, Carbone PP (1982) Toxicity and response criteria of the Eastern Cooperative Oncology Group. Am J Clin Oncol 5:649–655 7165009

[23] Eisenhauer EA, Therasse P, Bogaerts J, Schwartz LH, Sargent D, Ford R, Dancey J, Arbuck S, Gwyther S, Mooney M, Rubinstein L, Shankar L, Dodd L, Kaplan R, Lacombe D, Verweij J (2009) New response evaluation criteria in solid tumours: revised RECIST guideline (version 1.1). Eur J Cancer 45:228–247 19097774 10.1016/j.ejca.2008.10.026

[24] Morikawa H, Oba T, Kiyosawa N, Iji R, Amitani M, Chino T, Shimizu T, Ono M, Ito T, Kanai T, Maeno K, Ito KI (2023) Significance of skeletal muscle index-to-body mass index ratio as a predictor of post-surgical bleeding after mastectomy in patients with breast cancer. Breast Cancer 30:933–942 37440158 10.1007/s12282-023-01483-0

[25] Shachar SS, Deal AM, Weinberg M, Nyrop KA, Williams GR, Nishijima TF, Benbow JM, Muss HB (2017) Skeletal muscle measures as predictors of toxicity, hospitalization, and survival in patients with metastatic breast cancer receiving taxane-based chemotherapy. Clin Cancer Res 23:658–665 27489287 10.1158/1078-0432.CCR-16-0940 PMC5290138

[26] Meyer HJ, Dermendzhiev T, Hetz M, Osterhoff G, Kleber C, Denecke T, Henkelmann J, Werdehausen R, Hempel G, Struck MF (2024) Body composition parameters in initial CT imaging of mechanically ventilated trauma patients: single-centre observational study. J Cachexia Sarcopenia Muscle 15:2437–2446 39185615 10.1002/jcsm.13578 PMC11634470

[27] Fridman WH, Pagès F, Sautès-Fridman C, Galon J (2012) The immune contexture in human tumours: impact on clinical outcome. Nat Rev Cancer 12:298–306 22419253 10.1038/nrc3245

[28] Rier HN, Jager A, Sleijfer S, Van Rosmalen J, Kock MCJM, Levin M-D (2017) Low muscle attenuation is a prognostic factor for survival in metastatic breast cancer patients treated with first line palliative chemotherapy. Breast 31:9–15 27810702 10.1016/j.breast.2016.10.014

[29] Surov A, Strobel A, Borggrefe J, Wienke A (2023) Low skeletal muscle mass predicts treatment response in oncology: a meta-analysis. Eur Radiol 33:6426–6437 36929392 10.1007/s00330-023-09524-0

[30] Li ZH, Xu T, Zhang YJ, Jiang JH, Mi YZ, Li JX, Shen J, Fu YR, Qin BY, Lin F, Fu DJ, Yue MJ, Ma SM, Li QF (2024) Dynamic changes in body composition during XELOX/SOX chemotherapy in patients with gastric cancer. Front Oncol 14:1309681 38746684 10.3389/fonc.2024.1309681 PMC11091368

[31] Xu T, Li ZH, Liu T, Jiang CH, Zhang YJ, Li H, Jiang Y, Zhao J, Guo WJ, Guo JY, Wang L, Li JX, Shen J, Jin GW, Zhang ZW, Li QF (2022) Progress in research on antitumor drugs and dynamic changes in skeletal muscles. Front Pharmacol 13:893333 35873591 10.3389/fphar.2022.893333 PMC9298970

[32] Kodera Y (2015) More than 6 months of postoperative adjuvant chemotherapy results in loss of skeletal muscle: a challenge to the current standard of care. Gastric Cancer 18:203–204 24820695 10.1007/s10120-014-0381-z

[33] van der Meij BS, Teleni L, McCarthy AL, Isenring EA (2021) Cancer cachexia: an overview of diagnostic criteria and therapeutic approaches for the accredited practicing dietitian. J Hum Nutr Diet 34:243–254 33038282 10.1111/jhn.12811

[34] Sugawara K, Yamashita H, Urabe M, Okumura Y, Yagi K, Aikou S, Seto Y (2020) Poor nutritional status and sarcopenia influences survival outcomes in gastric carcinoma patients undergoing radical surgery. Eur J Surg Oncol 46:1963–1970 32402508 10.1016/j.ejso.2020.04.044

[35] Ding P, Lv J, Sun C, Chen S, Yang P, Tian Y, Zhou Q, Guo H, Liu Y, Zhao Q (2022) Combined systemic inflammatory immunity index and prognostic nutritional index scores as a screening marker for sarcopenia in patients with locally advanced gastric cancer. Front Nutr 9:981533 36046129 10.3389/fnut.2022.981533 PMC9421237

[36] Funahashi Y, Okamoto K, Adachi Y, Semba T, Uesugi M, Ozawa Y, Tohyama O, Uehara T, Kimura T, Watanabe H, Asano M, Kawano S, Tizon X, McCracken PJ, Matsui J, Aoshima K, Nomoto K, Oda Y (2014) Eribulin mesylate reduces tumor microenvironment abnormality by vascular remodeling in preclinical human breast cancer models. Cancer Sci 105:1334–1342 25060424 10.1111/cas.12488 PMC4462349

[37] Yoshida T, Ozawa Y, Kimura T, Sato Y, Kuznetsov G, Xu S, Uesugi M, Agoulnik S, Taylor N, Funahashi Y, Matsui J (2014) Eribulin mesilate suppresses experimental metastasis of breast cancer cells by reversing phenotype from epithelial–mesenchymal transition (EMT) to mesenchymal–epithelial transition (MET) states. Br J Cancer 110:1497–1505 24569463 10.1038/bjc.2014.80 PMC3960630

[38] Shimizu T, Oba T, Oshi M, Ito K-I (2024) Eribulin promotes proliferation of CD8+ T cells and potentiates T cell-mediated anti-tumor activity against triple-negative breast cancer cells. Breast Cancer Res Treat 203:57–71 37733186 10.1007/s10549-023-07111-x

[39] Neilson HK, Farris MS, Stone CR, Vaska MM, Brenner DR, Friedenreich CM (2017) Moderate-vigorous recreational physical activity and breast cancer risk, stratified by menopause status: a systematic review and meta-analysis. Menopause 24:322–344 27779567 10.1097/GME.0000000000000745

[40] Zhong S, Jiang T, Ma T, Zhang X, Tang J, Chen W, Lv M, Zhao J (2014) Association between physical activity and mortality in breast cancer: a meta-analysis of cohort studies. Eur J Epidemiol 29:391–404 24853250 10.1007/s10654-014-9916-1

[41] Garrone O, Paccagnella M, Abbona A, Ruatta F, Vanella P, Denaro N, Tomasello G, Croce N, Barbin F, Rossino MG, La Porta CAM, Sapino A, Torri V, Albini A, Merlano MC (2024) Moderate physical activity during neoadjuvant chemotherapy in breast cancer patients: effect on cancer-related inflammation and pathological complete response-the Neo-Runner study. ESMO Open 9:103665 39121813 10.1016/j.esmoop.2024.103665 PMC11364046

[42] Campbell TM, Campbell EK, Culakova E, Blanchard LM, Wixom N, Guido JJ, Fetten J, Huston A, Shayne M, Janelsins MC, Mustian KM, Moore RG, Peppone LJ (2024) A whole-food, plant-based randomized controlled trial in metastatic breast cancer: weight, cardiometabolic, and hormonal outcomes. Breast Cancer Res Treat 205:257–266 38446316 10.1007/s10549-024-07266-1 PMC11101531

