# Supplementary material for: Prognostic significance of baseline skeletal muscle index and its dynamics in patients with metastatic breast cancer undergoing eribulin treatment
Source: Breast Cancer Res Treat. 2025 Oct 15;214(3):419–29. doi: 10.1007/s10549-025-07827-y (PMC12583309; doi:10.1007/s10549-025-07827-y)
Supplement: Supplementary file 1 — Supplementary file1 (DOCX 39 KB) [file 10549_2025_7827_MOESM1_ESM.docx]

| Supplementary Table 1 Distribution of Changes in SMI and PNI | | | |
| --- | --- | --- | --- |
|  | PNI increase  (n = 20) | PNI decrease  (n = 35) | *p* value |
| SMI gain (n = 14) | 7 (50.0%) | 7 (50.0%) | 0.33 |
| SMI Loss/stable (n = 41) | 13 (31.7%) | 28 (68.3%) |  |
| PNI: prognostic nutritional index, SMI: skeletal muscle index | | | |
