# Supplementary material for: Prognostic significance of baseline skeletal muscle index and its dynamics in patients with metastatic breast cancer undergoing eribulin treatment
Source: Breast Cancer Res Treat. 2025 Oct 15;214(3):419–29. doi: 10.1007/s10549-025-07827-y (PMC12583309; doi:10.1007/s10549-025-07827-y)
Supplement: Supplementary file 2 — Supplementary file2 (PPTX 41 KB) [file 10549_2025_7827_MOESM2_ESM.pptx]

## Slide 1
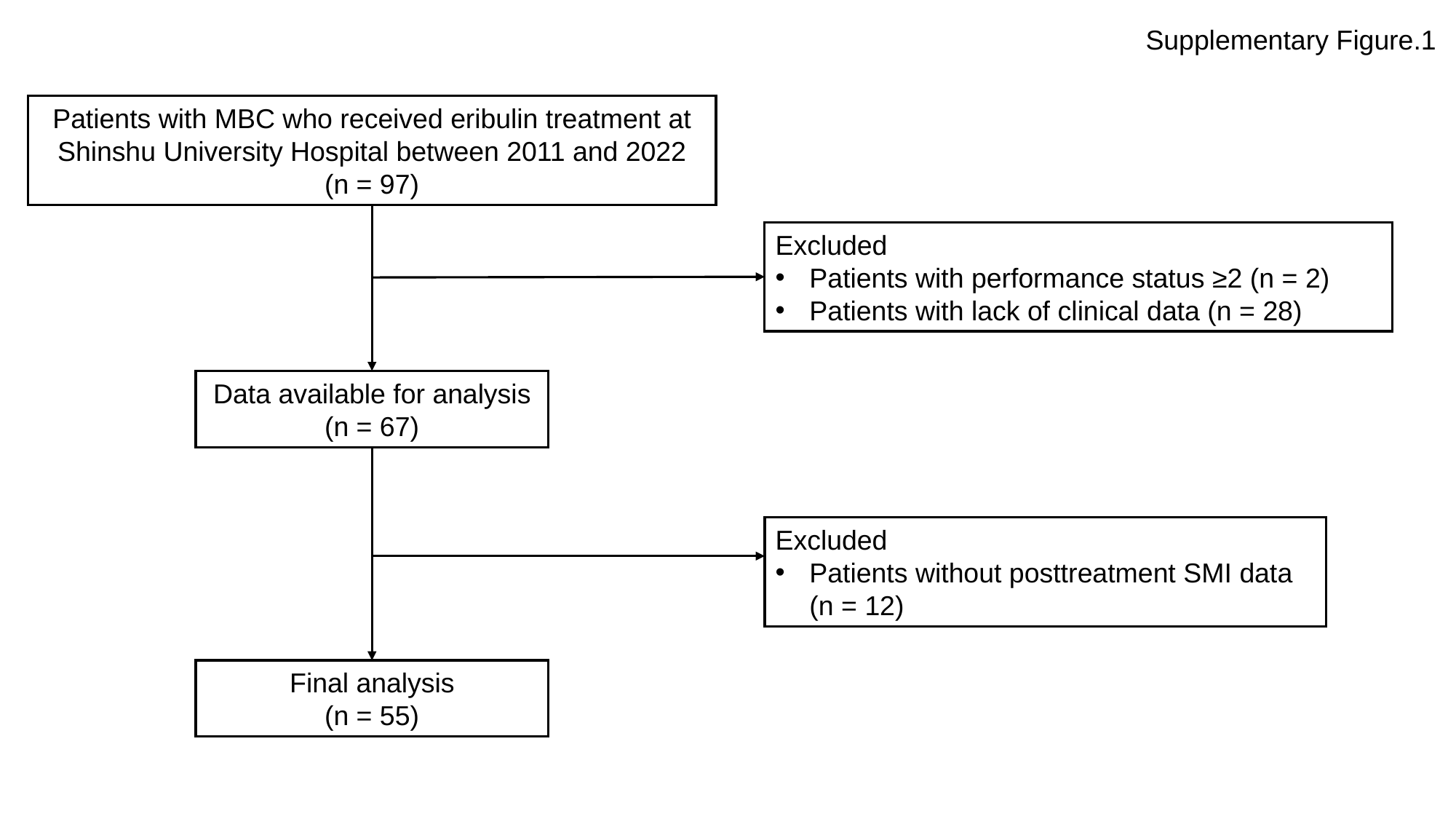

Supplementary Figure.1
Patients with MBC who received eribulin treatment at Shinshu University Hospital between 2011 and 2022
(n = 97)
Excluded
Patients with performance status ≥2 (n = 2)
Patients with lack of clinical data (n = 28)
Data available for analysis
(n = 67)
Excluded
Patients without posttreatment SMI data (n = 12)
Final analysis
(n = 55)
