# Supplementary figures and images for: Prognostic significance of baseline skeletal muscle index and its dynamics in patients with metastatic breast cancer undergoing eribulin treatment
Source: Breast Cancer Res Treat. 2025 Oct 15;214(3):419–29. doi: 10.1007/s10549-025-07827-y (PMC12583309; doi:10.1007/s10549-025-07827-y)

## Slide 1
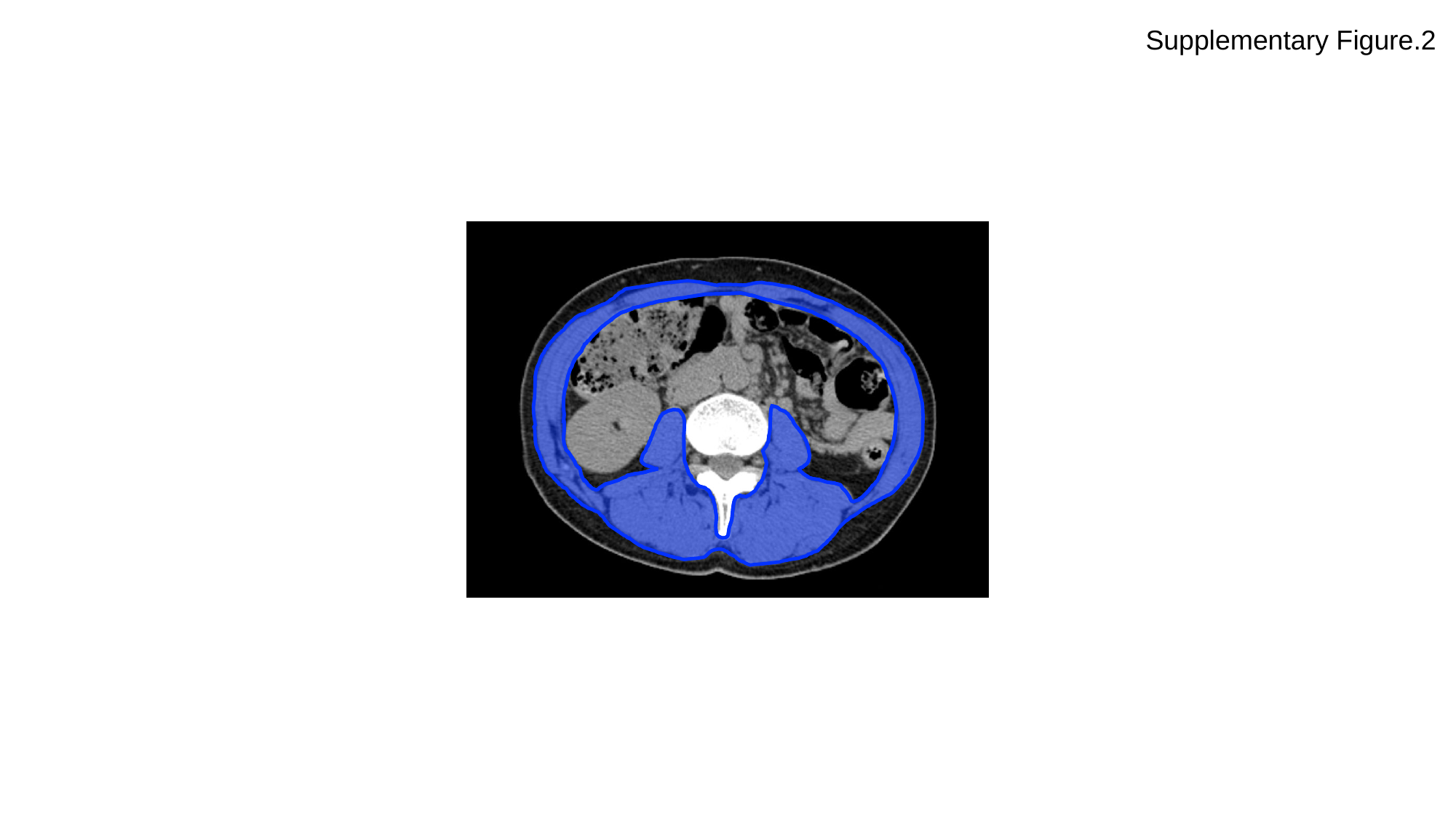

Supplementary Figure.2

Supplement: Supplementary file 3 — Supplementary file3 (PPTX 364 KB) [file 10549_2025_7827_MOESM3_ESM.pptx]

## Slide 1
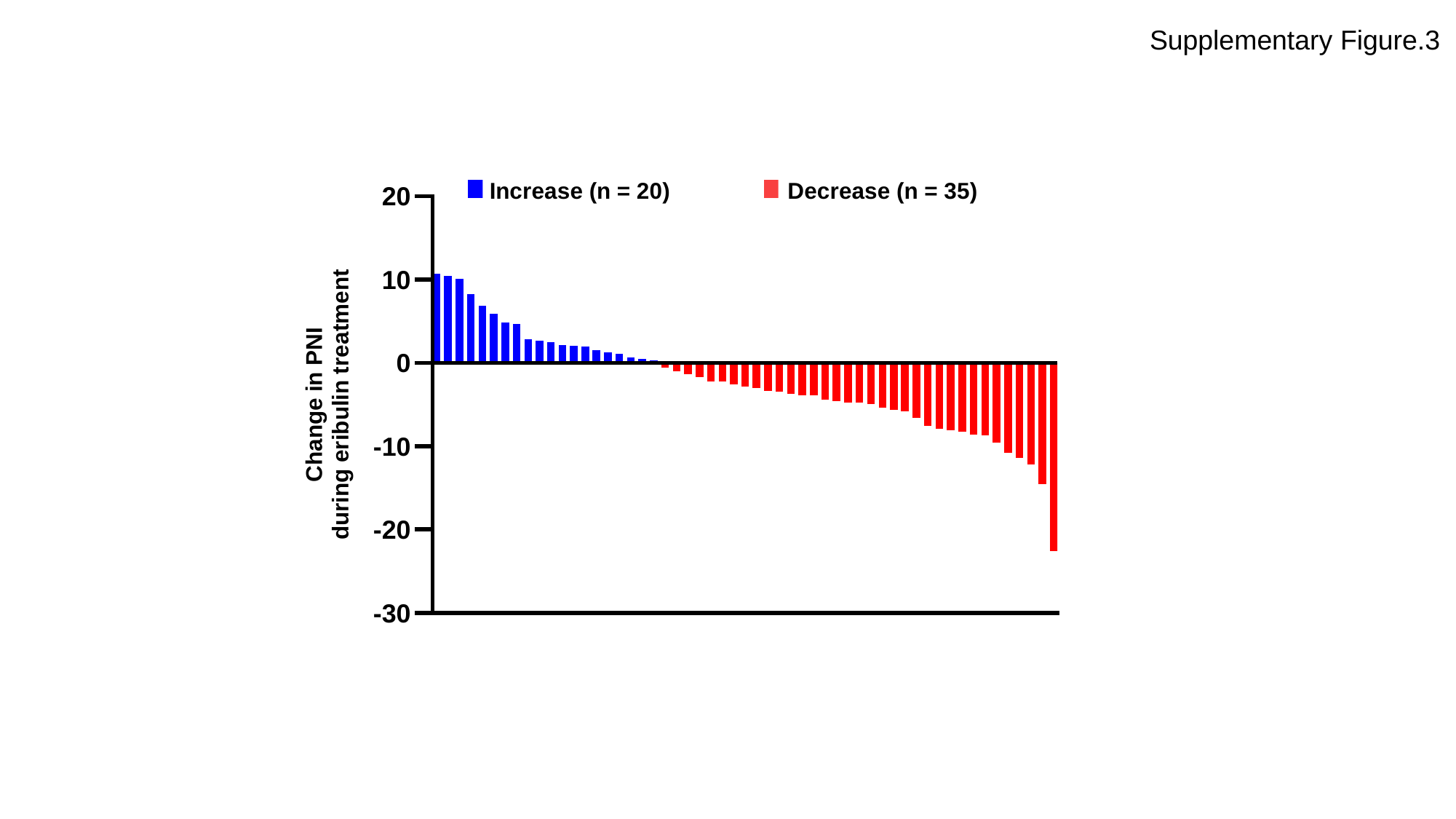

Supplementary Figure.3

Supplement: Supplementary file 4 — Supplementary file4 (PPTX 59 KB) [file 10549_2025_7827_MOESM4_ESM.pptx]
